# 761 Burn first aid in Australian pre-hospital environments

**DOI:** 10.1093/jbcr/irac012.314

**Published:** 2022-03-23

**Authors:** Bronwyn R Griffin, Roy M Kimble, Maleea Holbert

**Affiliations:** Griffith University, South Brisbane, Queensland; Children's Hospital Queensland, South Brisbane, Queensland; Children's Hospital Queensland, South Brisbane, Queensland

## Abstract

**Introduction:**

Best-practice burns first aid is well defined as 20 minutes of cool running water (CRW) within three hours of injury and an expectation of burn care in Australia. This study aims to identify barriers to applying this intervention and assess burn first aid knowledge amongst Australian paramedics.

**Methods:**

Using multiple methods we assessed; 1) burn first aid adequacy in a cross-sectional study of health care professionals, utilizing a prospectively collected registry of patients managed at an Australian tertiary children’s hospital. Logistic regression models were used to evaluate the relationship between first aid adequacy between health services (eg. Paramedics and emergency departments). Then 2) paramedics completed a questionnaire containing demographic and clinical expertise and environment as well as recording immediate first aid management across five multiple choice burn case scenarios

**Results:**

Overall, 31.3% of children received adequate CRW from caregivers. Factors associated with caregiver inadequacy of CRW were very young age and early adolescence (p< 0.001) rural location ( *P* = 0.045), low socioeconomic status ( *P* = 0.030) amongst others. Paramedics and general practitioners provided adequate cooling to 184/735 (25.0%) and 52/215 (24.2%) of their patients, respectively. Local general hospitals provided adequate CRW to 1019/1809 (56.3%) patients. Paramedic questionnaire responses (n=326) identified 56% of paramedics answered all burn case scenarios correctly. Respondents who treated a burn within six months scored higher on burn first aid scenarios compared to paramedics who had not recently treated a burn (p=0.004).

**Conclusions:**

: deficiencies remain in the cooling of paediatric burns patients at all levels of initial management. First aid delivery was significantly worse in children aged 0‐2, adolescents aged 15‐16, those living rurally, and the socioeconomically disadvantaged.

